# Factors influencing the approaches to studying of preclinical and clinical students and postgraduate trainees

**DOI:** 10.1186/1472-6920-11-22

**Published:** 2011-05-20

**Authors:** Dakshitha P Wickramasinghe, Dharmabandu N Samarasekera

**Affiliations:** 1 Department of Surgery, Faculty of Medicine, University of Colombo, Sri Lanka

## Abstract

**Background:**

Students can be classified into three categories depending on their approaches to studying; namely, deep approach (DA), strategic approach (SA) and surface apathetic or superficial approach (SAA). The aim of this study was to identify factors affecting the approaches to studying among Sri Lankan medical undergraduates and post graduate trainees and to analyze the change in the pattern of study skills with time and experience.

**Method:**

Pre-clinical and clinical students of the Faculty of Medicine, University of Colombo and postgraduate trainees in Surgery at the National Hospital of Sri Lanka were invited to complete the Approaches and Study Skills Inventory for Students (ASSIST) questionnaire.

**Results:**

A total of 187 pre clinical (M: F = 96:91), 124 clinical (M: F = 61:63) and 53 post graduate trainees (M: F = 50:3) participated in the study. Approaches of male and female students were similar. SA was significantly affected by age among the preclinical students (p = 0.01), but not in other groups. Among pre-clinical students, males preferred a teacher who supported understanding (p = 0.04) but females preferred a passive transmission of information (p < 0.001). This, too, was not visible among other groups. A linear regression performed on group (batch), gender, island rank at GCE Advance Level (AL) examination, self appraisal score and the preference scores of type of teacher only managed to explain 35% or less of variance observed for each approach in individual groups.

**Conclusion:**

Different factors affect the approach to studying in different groups but these explain only a small fraction of the variance observed.

## Background

Students can approach an academic task focussing on understanding or reproducing. This in fact, was the basis of a landmark study in 1976 by Marton and Saljo [1]. These ideas were followed up by Entwistle [2] and Ramsden [3] in 1981 and 1992 respectively. Subsequent work demonstrated that different approaches will affect the outcome of study programs [4-6] and that there are significant differences between the East and the West [7]. The notion that students can change their learning approach however has conflicting evidence [8-10].

There are 3 main approaches to studying; i.e. Deep approach (DA), Superficial (or surface apathetic) approach (SAA) and Strategic approach (SA). ). DA is an organised approach where the emphasis is internal and motivation comes from the relevance of the syllabus to their personal needs. SAA on the other hand, as the name itself implies, is superficial and includes memorisation and retrieval with unreflective associations. Learners with SA are mainly concerned about assessments and see studying as a game played to be won with various techniques like spotting potential questions from previous examination papers and making good personal impressions on the teachers.

The aims of this study were,

I. to identify the correlations between gender, age, self appraisal and preference for teacher type on the individual's approach to studying.

II. to analyze the change in the patterns of study skills, from a cross-sectional perspective.

## Methods

### Setting and participants

The study population is comprised of the first year (pre-clinical) and the final year (clinical) medical students and postgraduate trainees in General Surgery

There were a total of 364 participants, 187 from the preclinical (M: F = 96: 91), 124 from the clinical (M: F = 61: 63) and 53 post graduate trainees in Surgery (M: F = 50: 3).

For the undergraduates, the questionnaires were distributed at lectures. After explaining the purpose of the research, they were invited to complete and return the questionnaires. Postgraduates were approached individually during working hours and invited to participate in the study. All participants were assured confidentiality.

The undergraduate curriculum incorporates 5 streams which run parallel [11]; Introductory Basic Sciences Stream, Applied Sciences Stream, Clinical Sciences Stream, Behavioural Sciences Stream and Community Stream. Each academic year has 3 semesters and an examination at the end of each semester. The final pass mark is the cumulative total of results of all examinations held and the final MBBS examination.

The postgraduate training programme for registrars is coordinated by the Post Graduate Institute of Medicine (PGIM). They are first allocated to general surgical units for a year and later to speciality units for 2-3 monthly rotations.

The ethics committee of the Faculty of Medicine, University of Colombo (FMC) approved the study.

### The questionnaire

The participants were given the Approaches and Study Skills Inventory for Students (ASSIST) questionnaire. It is a revised version of the ASI developed by Entwistle and his colleagues at Lancaster University in the late 1970s and a product of the Enhancing Teaching-Learning Environments in Undergraduate Courses (ETL) team [12].

Respondents answer the questionnaire using 5 point modified Likert scales (*1 -disagree *and *5 - agree*). The first part includes 6 statements and deals with the respondent's perception of learning. The second part deals with the actual approaches to studying. This contains 52 statements combined into 13 subscales of four items each, which are then further grouped into the three main scales: DA, SA, and SAA. The third part containing 8 statements assesses the preference of course type and teaching methods and was answered using a like-dislike scale (1- definitely like, 5- definitely dislike). A final question asks the respondent to self evaluate himself/herself about previous assessments and was used as the self appraisal score used in the analysis. The English version of ASSIST has been validated by Byrne et al [13].

### Statistical analysis

This was a cross sectional study to test the hypothesis that the approaches to studying are different between undergraduates and post graduates.

The data were entered into a SPSS datasheet (SPSS Inc, Chicago, IL) and cumulative scores calculated as instructed in the ASSIST questionnaire. Each of the 52 statements was primarily attributed to one approach, and the cumulative score for each approach was the sum of the values of these statements.

The effect of group on each approach as well as preference of the type of teacher was analysed using the Kruskal-Wallis test (data not shown). Spearman correlation was used to assess the correlation between age and scores of each approach. The effect of gender was analysed using the Mann-Whitney test. Unless otherwise specified, analysis was done separately for each approach and group (e.g. -DA of pre-clinical, DA of clinical, etc). The relationships between the score of each approach with the other variables were analyzed using linear regression analysis. Significance level for the testing was <0.05, unless otherwise specified.

## Results

The questionnaire return rate was 99.4% for pre-clinical students, 68.8% for clinical students and 96.3% for post graduates and 86.0% cumulatively. The scores of each group for each approach are shown in table 1.

**Table 1:** The scores of each group for each approach

Approach		Deep Approach	Surface Apathetic Approach	Strategic approach
Group				
**Pre-clinical**	Median	62.00	47.00	67.00
**N = 187**	Minimum	41	15	39
	Maximum	77	72	82

**Clinical**	Median	57.00	52.00	58.50
**N = 124**	Minimum	16	16	17
	Maximum	82	72	81

**Post graduate in training**	Median	62.00	50.00	64.00
**N = 53**	Minimum	46	26	49
	Maximum	78	70	81

	Chi-square	32.794	15.643	66.617
				
**Kruskal Wallis test**	df	2	2	2
				
	Sig.	.000	.000	.000
			
Significance	Pre-clinical Vs. Clinical	P < 0.001	P < 0.001	P < 0.001
				
	Clinical Vs. PG	P < 0.001	P = 0.105	P < 0.001
				
	Pre-clinical Vs PG	P = 0.669	P = 0.075	P = 0.03

There was a significant correlation between DA and SA (ρ = 0.51, p < 0.001) and a small correlation between DA and SAA (ρ = 0.214, p < 0.001). There was no correlation between SA and SAA (ρ = 0.056, p = 0.143). In all three groups, SA had the highest median score, followed by DA. SAA had the lowest median. Pre-clinical and PG trainees had the highest mean for DA, while Clinical students had the highest mean for SAA and Pre clinical students for SA.

### Effect of age and gender

There were no statistically significant differences seen between male and female students of each group and the median scores of the approaches to studying (table 2).

**Table 2:** The effect of gender on approaches to studying

		DA		SAA		SA		TI		SU	
Pre Clinical	Mean (male, female)	61.9	60.8	46.4	47.5	66.2	66.4	14.5	16.0	16.0	15.5
	
	Mann-Whitney U	3950.5		4126.0		4237.0		3088.0		3743.5	
	
	Asymp. Sig. (1-tailed)	.130		.257		.362		0.0007		0.08	

Clinical	Mean (male, female)	55.7	55.7	51.7	51.8	57.1	57.1	15.7	15.7	14.9	15.9
	
	Mann-Whitney U	1822.500		1876.000		1869.000		1897.5		1671.0	
	
	Asymp. Sig. (1-tailed)	.310		.410		.397		0.12		0.2	

Post graduate	Mean (male, female)	62.1	59.6	49.7	40.3	64.6	67.0	12.2	11.7	16.6	16.0
	
	Mann-Whitney U	47.500		36.500		51.500		70.0		60.5	
	
	Asymp. Sig. (1-tailed)	.144		.069		.182		0.85		0.57	

**Figure 1:**
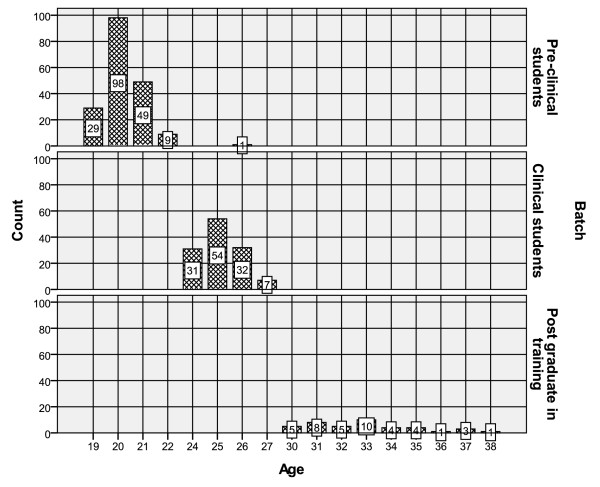
**Age distribution of the participants**.

There was a significantly positive correlation between age and SA among the preclinical students (ρ = 0.206, p = .002), but no other significant relationship was seen in other groups for any of the approaches (table 3) (figure 1).

**Table 3:** Effect of age on approaches to studying

		DA	SAA	SA
Preclinical	Correlation coeffieicient	-.033	.031	.206
	
	Sig.	= .657	.675	.005

Clinical	Correlation coeffieicient	.054	.108	.060
	
	Sig.	.549	.232	.509

PG	Correlation coeffieicient	.110	.078	.050
	
	Sig.	.494	.630	.755

Among pre clinical students, gender had a statistically significant effect on the preference of the type of teacher, where the males preferred a teacher who supported understanding [U = 3743.5, p = 0.04,] and females preferred a passive transmission of information [U = 3094.5, p < 0.001]. This difference was not visible among the clinical students [SU (U = 1671, p = 0.104), TI (U = 1897.5, p = 0.456] or the PG trainees [SU (U = 60.5, p = 0.318), TI (U = 70, p = 0.43)].

### Regression analysis

Multiple regression analysis was performed for each approach separately for undergraduate (i.e. pre-clinical and clinical students) and post graduates. Gender, self appraisal score and the preference scores of a teacher supporting understanding and transmitting information were the variables included. Island rank at AL examination was used as a variable only in undergraduates. This was because the postgraduates belonged to several AL batches and their island ranks would not provide a comparable result (Undergraduates - Table 4, Postgraduates - Table 5).

**Table 4:** Undergraduates

		DA			SAA			SA		
**Model summary**	R	0.505			0.395			0.608		
	
	Adjusted R square	0.236			0.135			0.353		

**ANOVA**	F	13.79			7.465			23.595		
	
	Sig.	<0.0001			<0.0001			<0.0001		

		**B**	**SE of B**	**β**	**B**	**SE of B**	**β**	**B**	**SE of B**	**β**
	
	(Constant)	46.130	4.908		28.450	5.973		50.305	5.293	
	
	Batch	-5.257	1.041	-.297	3.297	1.267	.163	-8.067	1.123	-.389
	
	Gender	-.505	.990	-.029	-.106	1.205	-.005	.817	1.068	.040
	
**Coefficients**	Island rank	-.001	.007	-.006	.012	.008	.088	.001	.007	.008
	
	Self appraisal	.222	.393	.034	-1.385	.478	-.183	2.248	.424	.290
	
	Supporting understanding	1.060	.178	.339	.505	.217	.141	.618	.192	.169
	
	Transmitting information	.197	.164	.068	.912	.199	.275	-.052	.176	-.015

DA - Deep approach

SAA - Superficial Apathetic Approach

SA - Strategic approach

**Table 5:** Postgraduates

		DA			SAA			SA		
**Model summary**	R	0.330			0.317			0.169		
	
	Adjusted R square	0.028			0.019			-0.060		

**ANOVA**	F	1.342			1.228			0.325		
	
	Sig.	P = 0.27			P = 0.313			P = 0.859		
		
		**B**	**SE of B**	**β**	**B**	**SE of B**	**β**	**B**	**SE of B**	**β**
	
	(Constant)	53.637	9.295		45.027	17.556		53.870	10.272	
	
**Coefficients**	Gender	-2.341	3.462	-.097	-9.193	6.539	-.203	2.691	3.826	.105
	
	Self appraisal	-.827	.948	-.138	-.994	1.791	-.088	.131	1.048	.021
	
	Supporting understanding	.716	.423	.246	.758	.799	.138	.419	.468	.136
	
	Transmitting information	.338	.235	.223	.616	.443	.217	.026	.259	.016

DA - Deep approach

SAA - Superficial Apathetic Approach

SA - Strategic approach

Among the undergraduates, there were statistically significant relationships between the group and supporting understanding with scores obtained for each approach. In addition self appraisal score was statistically significant for SAA and SA and the preference for a teacher who transmits information with SAA. The variables were able to predict 23% of variance of DA score, 13% of variance in SAA score and 35% of the variance of SA score.

There were no significant associations between any of the parameters analyzed and the preference of PG students. The models only explained 3%, 2% and <1% of DA, SAA and SA. (Table 5)

## Discussion

The results of the study indicate that the 3 groups essentially have different approaches to studying. In addition the correlation between age, gender, self appraisal and preference of a particular teaching type is also different between the groups. The roughly equal gender distribution is the norm seen at our medical school. Rather high response rate of preclinical students may be attributed to the free education system in Sri Lanka and students being grateful and cooperative. Lesser response rate from the clinical students may be because they are more occupied (i.e. clinical work and preparation for the final examination) than the preclinical students and this is understandable. The authors believe the PGs being individually approached resulted in the high return rate.

### Effect of the group

Of the 3 groups, the clinical students had the lowest score for DA. Though the decline in DA is surprising at first glance, this is a well described phenomenon in the West [14] but not in the East [15]. Previous articles that studied non-medical undergraduate and postgraduate trainees failed to identify any significant difference between them [16,17]. Our findings confirm this as far as preclinical students and PG students are concerned.

The increase in the superficial approach with the progression of the undergraduate course has been described previously in Australia [14] but not seen in a study conducted in Indonesia [15]. The pattern of scores of SA of the undergraduates in our study, correlates with the findings of Emilia et al [15] and that of preclinical students with PGs with the findings of Richardson et al [16]. We believe that the excessive workload of the undergraduate curriculum makes the clinical students adopt a superficial, less deep approach which may favour assessments.

### Effect of gender

The effect of gender in approaches to learning is a question that has intrigued generations. Our results failed to identify any significant difference, in keeping with the findings of the Indonesian study of Emilia et al [15] as well as other studies [18,19].

### Effect of age

The only significant observation in our study was the fact that, among the preclinical students, the SA increased with age and to the authors' knowledge, this is the first time this association is being reported. Though the correlation is small (ρ = 0.206), we feel it is important. Since AL examination is held only once a year the increase in age in the participants is accounted for by the increase number of attempts at the AL examination. This may suggest that students tend to be more strategic with the increased pressure of repeating the same examination which itself is probably the most competitive examination a doctor will have to sit in his life. The absence of this phenomenon among the clinical students could be due to the influence of the medical curriculum, since it has been at least 5 years since they sat for their AL examination. Both above factors suggest that this is a remnant effect of the AL examination. However it is important to remember that age and the group are confounders.

Work of Aaron et al [20] describes a different association of SAA and increased age. Another study done among medical students failed to identify any relationship between age and approaches to studying [16] but, studies done among business students [21] as well as students of degrees in psychology, sociology and social anthropology [22] describe an increase of DA with age, not SA as our results suggest.

### Preference of teacher types

The undergraduates preferred a teacher who "transmitted information" more than the postgraduates. This may have been brought on by the total transformation of teaching, where the undergraduate curriculum is mainly based on lectures, tutorials and discussions, and the postgraduate curriculum is mainly student centred and self motivated.

Our findings of the female preference of a teacher who passively transmits information is in keeping with the results of a study conducted by Severiens et al [19] also.

### Regression analysis

The associations between gender and group have been described above.

The island rank at AL examination (i.e. an indicator of examination performance) did not show a significant correlation for any approach. Even the present evidence on this issue is conflicting, with a review article that associates DA and SAA with better examination performance [23] and another that associates SAA with better performance at examinations [20]. However, studies done previously in Sri Lanka [24] as well as in Pakistan [25] have failed to demonstrate a similar performance benefit.

High self appraisal was negatively associated with the DA and SAA in pre-clinical students and positively with SA in both pre-clinical as well as clinical students. There were no associations seen with DA. Although there are no articles that deal with this association, Simon Cassidy in his article [26] describes a correlation between DA and self assessment skills. The absence of a relationship between self appraisal and DA together with the absence of an association between DA and examination performance, when considered in the light of the interpretations of Cassidy [26] could mean that a sub-factor of DA that correlates with examination performance and self appraisal may be missing in Sri Lankan students.

## Conclusions

The approaches to studying among Sri Lankan medical students are affected by the group and age (within the group) but not gender. The preference of the type of teacher is affected by the group and age and by gender among preclinical students. The findings suggest that characteristics of Sri Lankan students are some what different to students of other countries in the region. This is an important consideration in formulating the local curriculum. The decline in DA with progression in the medical school needs to be assessed and addressed by the curriculum developers. Perhaps a change in the frequency as well as the format of the assessments would be beneficial. It will also be worthwhile to improve the self-directed learning potential of undergraduate students.

There is no baseline value to compare the data for clinical students and post graduates and this is the main limitation of the present study. However, if the preclinical students are followed up and assessed at fixed intervals and if their approach as well as performance at examinations is assessed, perhaps more meaningful data could be obtained.

## Competing interests

The authors declare that they have no competing interests.

## Authors' contributions

All authors were involved in planning, data collection, analysis of data and writing the manuscript. All authors read and approved the final manuscript.

## Pre-publication history

The pre-publication history for this paper can be accessed here:

http://www.biomedcentral.com/1472-6920/11/22/prepub
